# The Effects of Competition on the Editorial Temper

**Published:** 1888-10-20

**Authors:** 


### The Effects of Competition on the Editorial Temper.

It is well known to the initiated that advertisements are the last things to come and the last to leave a newspaper. Many a journal has had its advertisement pages full long after its circulation has fallen to zero. For ten years, at least, the conductors of the *British Medical Journal*, as we recently shewed in an article on "Medical Journalism,"* have displayed such marked ability that they have distanced all rivals, and at the present time have the largest circulation of any medical journal in the English-speaking world. In Great Britain the circulation exceeds that of all other purely medical journals put together, and is more than double that of its chief competitor, the *Lancet*. This is, of course, very hard lines for the oldest medical paper, the decadence of which was stayed until about two years ago by the eminent ability of the late Dr. Wakley. On his death the pre-eminence of the *British Medical Journal* became more and more marked as the new editors displayed their calibre to an astonished profession. To add to the misfortunes of the *Lancet*, soon after Dr. Wakley's death The Hospital entered the field, and so placed our ancient contemporary between two fires, which seem to have told terribly upon its position and prospects. How terribly may be calculated when it is stated, that we have the best reasons for believing, The Hospital has a *bonâ fide* circulation in Great Britain to-day after only two years' existence which is far and away greater and more representative than that of the *Lancet* after fifty years of publication. Those who care to refresh their memories upon the subject of the attack on this Journal by the *Lancet* and to read the whole story from first to last, are referred to Vol. III. of The Hospital, No. 63, pages 180 and 183; No. 68, pages 261 and 262; No. 69, page 287; and No. 78, page 431; as well as to an article, entitled "The College of Physicians and the Medical Press," which appeared in the *Universal Review* of June last. We do not intend to again inflict the controversy upon our readers.

Fortunately, or unfortunately, owing possibly to the fact that the journalistic year of our contemporary is closing, and that the accounts once more bring home the facts of the situation with telling effect, the *Lancet* has thought proper to renew its attacks upon The Hospital this week by publishing letters from two members of the medical profession. These letters place our friends the writers in so unenviable a position that we, in sheer pity, refrain from comment. Here are the letters, both of which are addressed to the editors of the *Lancet*, and also the editorial comments thereon :—

THE MEDICAL PROFESSION AND POPULAR "MEDICAL" JOURNALISM.

Sirs,—I hear my name has been mentioned im connexion with the *Hospital* journal as a shareholder. I am much surprised at this, as last December, when I resigned my seat in the Council of the Hospitals Association, I gave orders for the disposal of the share, and was under the impression that this had been done I need hardly say I have taken steps to secure the legal withdrawal of my name if it has not already been accomplished.

I am, Sirs, yours obediently,
C. Theodore Williams.

Upper Brook Street, W., Oct. 11th, 1888.

Sirs,—My attention has just been called to the presence of my name in the list of persons holding shares of the "*Hospital*, Limited." I beg to inform you that I resigned my connexion with the Hospitals Association in May last, and with that I fully understood that my connexion with the *Hospital* paper would also cease.

I am, Sirs, yours obediently,
50, Harley Street, W., Oct. 11th, 1888. William Rose.

*** We feel sure that the explanation given above will be satisfactory to the authorities of the respective Colleges to which our correspondents belong, as well as to the profession generally. Indeed, it is impossible to believe that gentlemen of the professional standing occupied by the writer should have knowingly allowed their names to remain on the list of shareholders of such an enterprise as the *Hospital*—a journal which, starting avowedly as a lay publication, soon changed its front, and announced on its title-page "Medicine" as one of the subjects on which it proposed to instruct the public.—Ed. *L.*

* The Hospital, No. 68, Vol. III., pages 261 and 262.

We are informed that Dr. Williams and Mr. Rose remain shareholders because they have never executed a transfer of their holding nor communicated with the secretary on the subject. It was open for them to transfer their shares at any time if they thought well to do so, but until they have executed the legal instrument of transfer they must be aware that no one can remove their names from the register. It is amusing to see the assurance with which the Messrs. Wakley assume that at their dictation the councils of the Royal Colleges of Physicians and Surgeons of England will take upon themselves the function of regulating, selecting and controlling the investment of any money of which their fellows and members may be the fortunate possessors. Probably it would be a matter of surprise to either of the above gentlemen to find their investments, say in the London and North-Western Railway Company, had been disposed of without going through the form, at least, of obtaining their written assent, even had the Councils of the Royal Colleges of Physicians and Surgeons of England ordained it otherwise. Truly this beats the record of human folly. We are well content to leave our readers and the press to form their own opinion of this ridiculous attempt to excite prejudice by trying to revive an old controversy which was closed by Dr. Bristowe's courageous and noble acceptance of the presidency of The Hospitals Association, in relation to which this Journal does not hold any but the most independent, although friendly position. With an ever-growing circulation, the consistent support and encouragement of the press, which reproduces our articles and extracts in increasing quantity every week, and the consciousness that we have been enabled to do some substantial service to the public in many directions, we may well await with confidence that recognition by the business and advertising world which must soon make The Hospital one of the most prosperous and powerful of weekly journals.

It is with no little reluctance that we trouble the reader with these dry business facts, but it is, perhaps, well to exhaust the subject to-day. It may be stated then of our own circulation that a gentlemen, who is one of the most influential and trusted leaders of the medical profession, recently gave us the following amusing account of the career of his copy of The Hospital: It is first read by himself, then by his wife and family. He takes it on Mondays to the hospital, where his house-surgeon, the students, and the sister see it. After this it goes to the nurses in his wards, and in a recent instance, when he wanted to refer to a back number, on inquiry of the sister he found, that she had passed it on to the patients, and that one of them had been so interested that he begged to be allowed to take it home to show to his wife and friends. Besides the various classes of readers just enumerated, it is largely taken by secretaries, matrons, and hospital managers of every degree. It has also an increasing circulation in America, the Colonies, and on the continent of Europe. Such facts as these must tell upon the advertising department of a paper like the *Lancet*, for advertisers are beginning to recognise the folly of burying their advertisements in columns of similar announcements, which may never be read through, and are seldom referred to even by the few. It is necessary that an advertisement should be widely perused if it is to be profitable, and the manager of this journal frequently receives letters from advertisers in our columns, stating that the answers they receive are so numerous as to make it difficult to deal with them. We commend these facts to the consideration of those whom they may concern. The action of the present Editors of the *Lancet*, who, be it always remembered, are also its proprietors, proves once more, *Quos Deus vult perdere, prius dementat.*

